# Chaperone Sigma1R and Antidepressant Effect

**DOI:** 10.3390/ijms21197088

**Published:** 2020-09-25

**Authors:** Mikhail V. Voronin, Yulia V. Vakhitova, Sergei B. Seredenin

**Affiliations:** Department of Pharmacogenetics, FSBI “Zakusov Institute of Pharmacology”, Baltiyskaya Street 8, 125315 Moscow, Russia; juvv73@gmail.com

**Keywords:** Sigma1R chaperone, depression, antidepressants, BDNF, NGF, calcium signaling, ER stress, unfolded protein response, epigenetic regulation

## Abstract

This review analyzes the current scientific literature on the role of the Sigma1R chaperone in the pathogenesis of depressive disorders and pharmacodynamics of antidepressants. As a result of ligand activation, Sigma1R is capable of intracellular translocation from the endoplasmic reticulum (ER) into the region of nuclear and cellular membranes, where it interacts with resident proteins. This unique property of Sigma1R provides regulation of various receptors, ion channels, enzymes, and transcriptional factors. The current review demonstrates the contribution of the Sigma1R chaperone to the regulation of molecular mechanisms involved in the antidepressant effect.

## 1. Introduction

The incidence of depressive disorders is growing steadily and represents an acute medical and social problem [1]. Antidepressants used in clinical practice have proved their efficacy in long-term treatment [2]. However, such medication is accompanied by various side effects [3], and about one-third of patients do not achieve remission [4]. The development of new treatment options for depressive disorders becomes possible due to numerous studies of their pathogenesis. The monoamine hypothesis of depression was among the first proposed and was based on the efficiency of drugs that increase the levels of 5-HT or a combination of catecholamines in the synaptic cleft [5,6]. Recent studies suggest that a convergence of different molecular mechanisms may be associated with depressive disorders [7]. There is a growing body of evidence supporting the neurotrophin theory of depression, according to which the major role that impaired BDNF/trkB signaling in the hippocampus and prefrontal cortex plays in depression [8,9]. The contribution of inflammation, activation of microglia, and lipid peroxidation (LPO) processes on the pathogenesis of depression have also been revealed [10,11,12,13,14]. The role of glutamatergic processes in the development of depressive disorders and rapid antidepressant action has been confirmed experimentally [15,16,17]. The importance of potassium channels, intracellular calcium, and post-receptor signaling pathways has been demonstrated [18,19,20]. In the 1990s, sigma-1 receptors (Sigma1R) were considered as a pharmacological target for antidepressants. A prerequisite for these studies was the discovery of the affinity of antidepressants from the group of 5-HT reuptake inhibitors (SSRIs) for Sigma1R [21]. The antidepressant effect of most SSRIs is associated with a high affinity for the sodium-dependent serotonin transporter (SERT); however, no similar association with the affinity for Sigma1R was demonstrated [21,22,23,24,25]. At the same time, the ability of selective antagonists of Sigma1R to block the rapid and delayed antidepressant-like action of fluvoxamine, venlafaxine, and endogenous and exogenous agonists of Sigma1R, after a single administration, has been shown [26,27,28,29,30,31]. An attempt to introduce selective Sigma1R agonists into clinical practice as antidepressant drugs was unsuccessful [32,33,34]. Despite these findings, discoveries in molecular biology have revealed three important properties of Sigma1R: chaperone activity aimed at a large number of proteins, intracellular translocation within lipid microdomains, and interaction with a large number of chemical compounds [35,36,37]. Therefore, ligand regulation of Sigma1R may be a promising strategy for the optimization of pharmacotherapy of depressive disorders. This review aims to highlight the role of the chaperone Sigma1R in the pathogenesis of depression and the pharmacodynamics of antidepressants.

## 2. Structure and Functional Activity of the Chaperone Sigma1R

Sigma1R was first identified in the Tsung Ping Su laboratory in 1982 [38]. To date, the Sigma1R chaperone activity has been established, and a significant body of scientific data on the structure, functional activity, and ligand regulation of Sigma1R has been collected. Most of the studies are systematized and presented in detailed reviews [35,36,39,40,41,42,43,44]. The human, murine, rat, and guinea pig Sigma1R protein comprises 223 amino acid residues (~25 kDa) that are more than 90% identical. Sigma1R has a unique amino acid sequence and has no homology with known mammalian proteins [43,44]. In 2016 the crystal structure of a protein with one transmembrane domain for each monomer was determined under the general supervision of Andrew C. Kruse [45,46]. Sigma1R oligomerization affects the chaperone functional activity and depends on the interaction with ligands [41,47,48,49,50].

Chaperone Sigma1R is expressed in certain regions of the rodent brain, including the cortex and hippocampus [51,52,53,54,55]. The data obtained in laboratory animals are consistent with the distribution of Sigma1R in the human brain [56]. Sigma1R is a resident protein of the endoplasmic reticulum (ER) and is predominantly localized in the cholesterol-rich region of ER mitochondria-associated membranes (MAM) [35,40,57,58]. In this compartment, Sigma1R acts as a chaperone to stabilize IP_3_R3, maintaining Ca^2+^ flow from the ER to mitochondria and ATP production [59]. Chaperone Sigma1R acts ligand-dependently on ER Ca^2+^ sensor STIM1 and regulates store-operated Ca^2+^ entry [60]. The chaperone interaction with the VDAC2 channel influences the uptake of cholesterol and the synthesis of pregnenolone in mitochondria [61,62]. Sigma1R stabilizes the ER stress sensor IRE1, thereby prolonging its dimerization and promoting endonuclease activity and the production of a functionally active transcription factor, XBP1, which induces the expression of genes for neurotrophins, antioxidant defense proteins, and chaperones [63,64]. Sigma1R is able to form a Ca^2+^ sensitive complex with the main ER chaperone BiP (GRP 78, HSPA5) [59,65], which dissociates under the action of Sigma1R agonists, activating BiP [59,66]. The interaction of BiP and ER stress sensors IRE1, PERK, and ATF6 inhibits their activity [67]. In turn, without the ER stress and in combination with IRE1, BiP itself acts as an ER stress sensor and does not perform its normal chaperone functions [68]. The activity of Sigma1R in the MAM region is significant for the response to ER stress (UPR, unfolded protein response) in pathological conditions [40,69]. The interaction of Sigma1R with Rac1-GTPase influences the redox processes in neurons and the formation of dendritic spines [70,71]. The involvement of the chaperone in the regulation of p35 protein metabolism (CDK5 activator 1) is of paramount importance in axon elongation [72].

Sigma1R is involved in the formation of ER lipid compartments. During ligand activation or under conditions of cellular stress, the chaperone, as part of lipid microdomains, is capable of both redistribution within the ER and translocation into the region of the plasma and nuclear membranes [66,73,74]. Sigma1R engages in protein‒protein interactions and regulates the functional activity of G-protein coupled receptors (dopamine D_1_ and D_2_, opioid µ, cannabinoid CB_1_), tyrosine kinase receptor for neurotrophins trkB, receptor for platelet growth factor PDGFRβ, ion channels (ASICs, K_V_1.2, K_V_1.3, K_V_2.1, Na_V_1.2, and GluN1), and other plasma membrane proteins [35,39,41]. The interaction of Sigma1R with emerin on the nucleus inner membrane provides the formation of the chromatin remodeling protein complex, its interaction with the Sp3 protein, and regulation of the transcription of target genes [35,75]. The client proteins of the Sigma1R chaperone are involved in the pathogenesis of depressive disorders [76,77,78,79,80,81,82,83], which indicates the importance of Sigma1R for the pharmacodynamics of antidepressants. The effects of Sigma1R on proteins are not limited to experimentally confirmed chaperone interactions. For example, upon ligand activation, Sigma1R enhances the expression of subunits (GluN2A, GluN2B) and the traffic of NMDA receptors to the plasma membrane of neurons [84] and regulates the activity of various types of Ca^2+^ channels [85,86].

Thus, upon ligand activation, chaperone Sigma1R is capable of translocation between intracellular compartments and interactions with client proteins expressed in the brain and involved in the pathogenesis of depressive disorders.

## 3. Antidepressant Activity of Sigma1R Ligands

Sigma1R has a relatively low molecular weight and the ability to bind with many chemical substances and psychotropic drugs [37,39,41,87]. This aspect suggests the presence of specific binding sites that evolved during the interaction with endogenous and exogenous compounds [43,88].

Table 1 presents Sigma1R ligands with established antidepressant activity. SSRIs fluvoxamine and sertraline have the highest affinity for Sigma1R. On the other hand, lower affinity is shown for tricyclic antidepressant imipramine and SSRIs (±)-fluoxetine, escitalopram, and citalopram [21,23]. SSRIs are considered to act as Sigma1R agonists [89,90]. Nevertheless, studies in *Sigmar1* knockout (*Sigmar1*^−/−^) mice indicate the possibility of SSRIs to act as inverse agonists or antagonists of Sigma1R [91,92], which is consistent with the results of in vitro studies (Table 1) [23,93,94].

The noncompetitive NMDA receptor antagonist ketamine induces a rapid antidepressant effect [17]. Ketamine racemate displaces the labeled prototype Sigma1R agonist (+)-pentazocine from the binding sites with micromolar Ki value [101] (Table 1). However, in experiments with Swiss Webster mice, the Sigma1R antagonists BD-1047 and NE-100 did not affect the antidepressant-like action of ketamine (40 mg/kg, i.p.) in the forced swim test [101].

Plausible endogenous ligands for Sigma1R are certain neurosteroids, such as dehydroepiandrosterone (DHEA), dehydroepiandrosterone sulfate (DHEAS), pregnenolone sulfate (PREGS), and progesterone [106,107,113]. DHEA (*Ki* = 2.96 μM) (Table 1), DHEAS (*Ki* = 15.13 μM), and PREGS (*Ki* = 3.2 μM) show agonistic properties, whereas progesterone (*Ki* = 175 nM) is characterized as a Sigma1R antagonist [106,114,115]. Notably, neurosteroids with Sigma1R agonist properties demonstrate antidepressant-like activity [28,105,108,116,117], while progesterone prevents their effects [28,116]. DHEA has been shown to possess antidepressant effects when administered to patients with depressive disorders [118].

Molecular docking and targeted mutagenesis studies, as well as rigorous structure/affinity relationship analysis, made it possible to establish an adequate Sigma1R pharmacophore model for selection or design of selective ligands [41,42]. Selective agonists igmesine (JO-1784, CL-1019) [119] and cutamesine (SA4503) [120] have antidepressant effects (Table 1) [32,33,34]. In preclinical studies, antidepressant-like activity was shown for prototype benzomorphic ligands ((+)–SKF-10.047, (+)-pentazocine) and the selective agonist PRE-084 [26,30,108,121,122,123,124,125,126,127]. In the vast majority of studies, antidepressant-like action was observed within 1 h after drug administration. On the contrary, the available data show no independent effect of selective Sigma1R antagonists (BD-1047, BD-1063, NE-100) on the behavior of experimental animals in depression models upon administration. However, antagonists interfered with the antidepressant-like action of Sigma1R ligands with agonist properties [26,27,28,29,92,101,102,105,108,116,127,128,129,130,131].

Despite the lack of an established relationship between the efficacy of antidepressants and Sigma1R affinity, the use of Sigma1R antagonists in vivo and in vitro demonstrates the involvement of the protein in pharmacodynamics of antidepressants.

## 4. The Role of *SIGMAR1* Gene Activity in the Pathogenesis of Depression and Pharmacodynamics of Antidepressants

*Sigmar1* knockout mice exhibit depression-like behavior in the forced swim test [91,132,133,134,135] and the tail suspension test [133,134,135]. In knockout males, the immobilization time decreased faster than in females [91,133]. It is known that the antidepressant-like effect of the selective agonist Sigma1R igmesine (40 mg/kg, i.p.) [119,122] did not develop in *Sigmar1*^−/−^ mice [91,92]. The effect of tricyclic antidepressants on the duration of immobilization in the forced swim test was similar in *Sigmar1*^−/−^ mice and wild-type mice [91]. However, in *Sigmar1*^−/−^ mice, SSRIs with high affinity for Sigma1R, sertraline, and fluoxetine at a dose of 40 mg/kg, i.p. reduced the duration of immobilization compared to wild-type mice [21,91,92].

Chronic stress and activation of the hypothalamic–pituitary–adrenal (HPA) axis plays an important role in the etiology of depressive disorders [136,137,138,139]. Indeed, *Sigmar1*^−/−^ mice responded to acute mild restraint stress with increased HPA axis activity and increased CRF expression. The levels of phosphorylation of glucocorticoid receptors and PKC were shown to decrease in the paraventricular nucleus of the hypothalamus [134].

Morphological changes and impaired synaptic plasticity in the hippocampus are markers of the severity of depressive disorders [140,141]. In this regard, a decrease in long-term potentiation (LTP) in the CA1 region of the hippocampus of *Sigmar1*^−/−^ mice should be noted [142]. Mutations in the *SIGMAR1* gene rs387906829 (c.304G>C, p.Glu102Gln) and rs796065352 (c.151+1G>T, c.92_151del, p.Gly31_Ala50del) cause a decrease in the number of mushroom spines in hippocampal neurons [143,144].

Clinical studies revealed an association of the rs1800866 nucleotide substitution (c.5A>C, A61C, p.Gln2Pro) in the *SIGMAR1* gene and major depressive disorder (MDD) in the Japanese population. The minor C allele has been shown to be less common in MDD patients [145]. Later, an association of this polymorphism with bipolar disorder in the Korean population was revealed [146]. These data indicate the role of *SIGMAR1* chaperone gene mutations in the development of depression.

## 5. Contribution of Sigma1R Chaperone to the Molecular Mechanisms of Depressive Disorders and the Pharmacodynamics of Antidepressants

### 5.1. Neurotrophins

The neurotrophin theory of depression is supported by new experimental evidence [9,147,148]. Despite the well-known contradictions of the research results [149], an important role of BDNF in the pathogenesis of depression and the development of both rapid and delayed antidepressant actions has been established [8,150]. In particular, decreased BDNF levels and impaired neuroplasticity in the hippocampus, prefrontal cortex, and amygdala have been demonstrated in rodent models of depression [151,152,153,154]. Chronic stress causes downregulation of BDNF and NGF in the murine hippocampus [155,156]. BDNF and NGF alone are capable of inducing an antidepressant-like effect [157,158,159]. Antidepressants are known to enhance the expression of neurotrophin genes [151,152,154,156,160].

#### 5.1.1. BDNF

Numerous in vitro and in vivo studies have shown the relationship between Sigma1R regulation and BDNF synthesis and release. In these studies, selective Sigma1R agonist SA4503 (1.0 μM) increased BDNF secretion in rat neuroblastoma B104 cells when incubated for up to nine days under serum deprivation. Seven days of incubation with the selective antagonist Sigma1R NE-100 (1.0 μM) abolished the effect of SA4503. The authors noted that, unlike a number of antidepressants that enhance BDNF mRNA production, SA4503 facilitated the post-translational processing of BDNF, which was associated with the neurotrophin secretion [161]. The contribution of Sigma1R to the regulation of BDNF secretion was revealed in the CCF-STTG1 astrocytoma cell line. Agonists (+)-SKF-10.047 (100 μM) and 4-PPBP (10 μM) increased BDNF secretion. The selective antagonist Sigma1R BD–1063 (15 nM) significantly reduced the effect of agonists [162].

In in vivo experiments, a two-week course of the selective Sigma1R agonist SA4503 at a dose of 1.0 mg/kg induced an increase in the BDNF content in the hippocampus, but not in the frontal cortex, of Wistar rats. However, single and chronic four-week administration of the compound (0.3 and 3.0 mg/kg, i.p.) did not affect the BDNF level in these brain regions [163].

According to Lenart et al., BDNF was involved in the Sigma1R-dependent mechanisms of action of fluvoxamine (20 mg/kg, p.o.) when it was administered for two weeks to Wistar rats with comorbid depression-like disorder in a model of streptozotocin diabetes [31]. Experimental animals were characterized by reduced BDNF level in blood serum; moreover, decreased levels of proBDNF, mBDNF, and Sigma1R proteins were found in the hippocampus and prefrontal cortex. Fluvoxamine, orally administered, normalized proBDNF, mBDNF, and Sigma1R levels. Abolishment of the antidepressant-like action of fluvoxamine by the selective antagonist Sigma1R NE-100 (1.0 mg/kg, p.o.) was accompanied by the elimination of the fluvoxamine effect on the content of proBDNF, mBDNF, and Sigma1R [31]. Institute of Cancer Research (ICR) outbred mice with cardiac dysfunction caused by aortic banding and a high-salt diet exhibited depression-like behavior in the tail suspension test, which was accompanied by a decrease in the level of the Sigma1R protein in the brain. Chronic intracerebroventricular infusion of the selective Sigma1R agonist PRE-084 restored the Sigma1R level to the control values [164]. It should be noted that, in these studies, Sigma1R ligands (fluvoxamine, NE-100) did not affect the BDNF mRNA level. These data are consistent with the action of the selective Sigma1R agonist SA4503, which, as mentioned above, promoted post-translational processing and secretion of BDNF in vitro [161].

Sigma1R activation mediates morphological and functional cellular responses by regulating trkB activity and associated signaling pathways. Thus, the selective agonist PRE-084 causes an increase in neurite outgrowth in the culture of cerebellar granular neurons due to the phosphorylation of trkB receptors. This action of PRE-084 is blocked by the selective Sigma1R antagonist BD-1063 [165].

Incubation of a primary culture of murine cerebral cortex neurons with imipramine or fluvoxamine in the concentration range of 0.1–10 μM enhances BDNF-induced glutamate release and increase of intracellular Ca^2+^ [100]. Analysis of the activation of BDNF signaling pathways upon incubation with imipramine revealed stimulation of PLCγ binding to trkB and p-PLCγ production in the absence of changes in the levels of p-trkB, p-Akt, p-ERK1, p-ERK2. BDNF-stimulated glutamate release was completely eliminated by the PLCγ1 inhibitor U73122 [166]. Antidepressants have been shown to potentiate the BDNF-evoked glutamate release and Ca^2+^ increase, which were blocked by U73122 and the IP_3_R1 antagonist xestospongin C [100]. Pre-incubation of cells with Sigma1R antagonist BD-1047 for 30 min followed by imipramine treatment prevented BDNF-dependent PLCγ phosphorylation and glutamate release. On the contrary, overexpression of the *Sigmar1* gene promoted these processes, which indicates the contribution of Sigma1R to the regulation of BDNF signaling cascades [100]. The mechanism of regulation of BDNF-dependent processes may be associated with the ability of Sigma1R to bind and transport cholesterol during ligand activation due to the presence of cholesterol recognition or interaction of amino acid consensus sequences (CRAC/CARC) [66,73,167]. Pilot studies have shown that CRAC/CARC motifs are present in the trkB sequence [168], and cholesterol itself is able to stimulate trkB phosphorylation [169]. The activity of both Sigma1R and trkB is associated with their localization in cholesterol-rich lipid domains [73,170,171,172,173], while Sigma1R ligands fluoxetine and escitalopram have the ability to accumulate in lipid rafts [174]. Ketamine, but not other NMDA antagonists, mediates the rapid redistribution of Gα_s_ from lipid rafts and the enhancement of cAMP synthesis [175]. The recent data have shown that SSRIs, imipramine, and ketamine bind to CARC motif in the trkB [169]. Therefore, when Sigma1R is activated by antidepressants or agonists in adjuvant therapy, not only a chaperone effect on trkB is possible [165], but also a positive effect on the binding of trkB to antidepressants due to cholesterol translocation to the CARC motifs region. It is of interest that pravastatin is capable of abolishing the effect of BDNF on the interaction between trkB and PLCγ [169]. The results of these studies correspond to the ability of Sigma1R both to participate in the transport and distribution of lipids [176] and to facilitate BDNF-dependent phosphorylation of PLCγ [100].

Nakano et al., using PC12 cells, experimentally proved the contribution of Sigma1R in the activation of the PI3K/Akt signaling pathway following fluvoxamine or DHEAS treatment [177]. Fluvoxamine at concentrations of 10 and 100 μM after 40 min of incubation under serum deprivation increased Akt1 phosphorylation at the Ser473 by more than 2- and 3.5-fold, respectively. Compared to fluvoxamine, BDNF was effective in just 2.5 min. Incubation of cells with DHEAS for 24 h caused a twofold increase in the p-Akt1 level (Ser473) [177]. These data are consistent with the results obtained in olfactory bulbectomy mouse model of depression [105,178]. The depression-like behavior in olfactory bulbectomized mice is accompanied by a decrease in neurogenesis in the hippocampal dentate gyrus and inhibition of phosphorylation of trkB and CaMKIIα (Thr286), PKCα (Ser657), Akt (Ser473), Akt (Thr308), ERK, and CREB (Ser133). Chronic administration of DHEA (30 and 60 mg/kg, p.o.) restored the levels of above-mentioned proteins dose-dependently up to control values, while injection of NE-100 (1.0 mg/kg, p.o.) 30 min before DHEA prevented the effect of the neurosteroid [104,105].

BDNF has a regulatory effect on the glycogen synthase kinase 3β (GSK-3β) through the activation of trkB [179]. GSK-3β is involved in the process of neuroplasticity, contributes to the pathogenesis of depressive disorders, and plays a crucial role in the regulation of cellular homeostasis. The major substrates of GSK-3β are transcription factors, which transduce the effect of enzyme activation on gene expression [180,181,182,183]. GSK-3β is characterized by complex regulatory mechanisms [182]. Phosphorylation of the enzyme at the Ser9 inhibits the activity of GSK3B with respect to prephosphorylated substrates; however, modification of other proteins may persist [182,184]. In a number of experiments, the relationship between Sigma1R activation and GSK3B regulation was established; in particular, selective agonist Sigma1R PRE-084 increased the level of pGSK-3β (Ser9) in the hippocampus of naïve C57Bl/6 mice [127]. It is noteworthy that the serotonin and norepinephrine reuptake inhibitor venlafaxine, which has no affinity for Sigma1R, produces a similar effect [23,127]. In vitro experiments established the contribution of the trkB and activation of the Akt-regulated signaling pathway to the Sigma1R-dependent augmentation of GSK-3β phosphorylation [127]. However, the depression-like behavior of olfactory bulbectomized mice was accompanied by an increase in the pGSK-3β (Ser9) in the hippocampal dentate gyrus [105]. Endogenous Sigma1R agonist DHEA at a dose of 60 mg/kg, p.o. reduced the level of pGSK-3β (Ser9) in experimental animals to control values. The dependence of inhibitory phosphorylation of GSK-3β on Sigma1R was subsequently confirmed by preliminary administration of the Sigma1R antagonist NE-100 (1.0 mg/kg, p.o.), which mitigated the effect of DHEA [105]. Considering chaperone Sigma1R significance for the UPR process [59,63,185,186], it is possible that this association reflects the induction of UPR processes by an agonistic effect on Sigma1R. This assumption is consistent with the established decrease of pGSK-3β (Ser9/21) and an increase in the enzyme activity upon UPR activation in neuroblastoma cells [187]. On the other hand, it was demonstrated that activation of astrocytes induced by the Sigma1R agonist (+)–SKF-10.047 was accompanied by an increase in GSK-3β phosphorylation at Ser9 [188]. Additional studies are required to establish the patterns and significance of interaction between Sigma1R, BDNF, and GSK-3β in the pathogenesis of depressive disorders.

**Figure 1:**
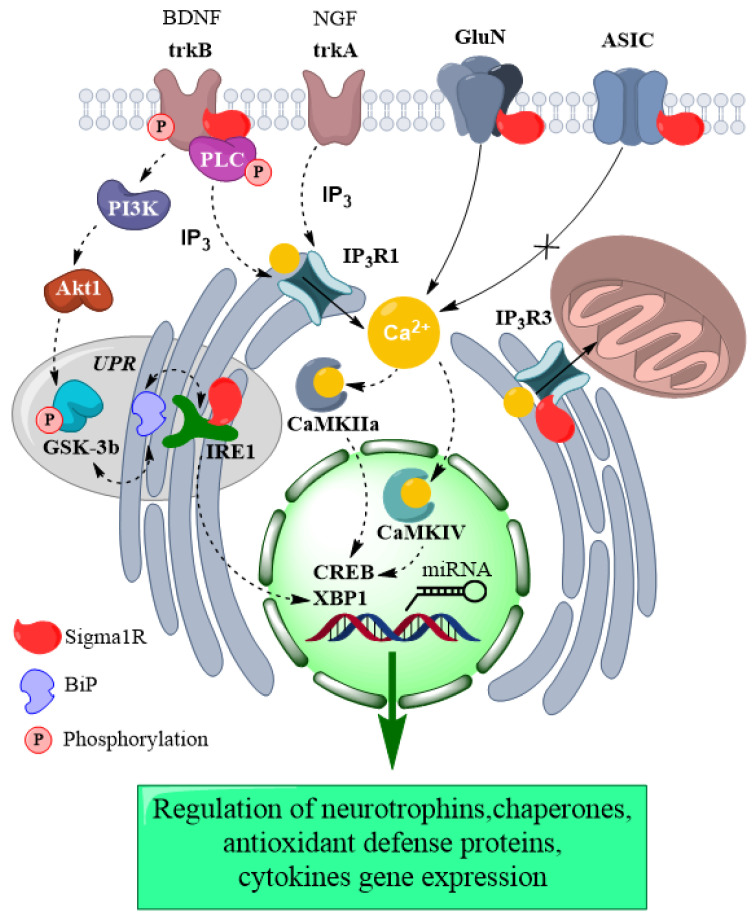
The contribution of Sigma1R activation in intracellular mechanisms of the antidepressant effect. The figure represents chaperone interactions of Sigma1R and functional consequences on proteins triggered by Sigma1R activation. Interaction of Sigma1R with BDNF receptor, GluN, Ca^2+^ channels, and ER stress sensor promotes activation of signal cascades and Ca^2+^-dependent proteins. These processes jointly with Sigma1R-dependent epigenetic regulation cause changes in expression of genes of neurotrophins, chaperones, proteins of antioxidant defense, and cytokines that provide the antidepressant effect. Plain arrow—Ca^2+^ flux; dashed arrow—Sigma1R-mediated influences. Akt1—RAC-alpha serine/threonine-protein kinase; ASIC—Acid-sensing ion channel; BDNF—Brain-derived neurotrophic factor; BiP—Endoplasmic reticulum chaperone BiP; CaMKIIα—Calcium/calmodulin-dependent protein kinase II alpha; CaMKIV—Calcium/calmodulin-dependent protein kinase type IV; CREB—Cyclic AMP-responsive element-binding protein; GluN—Glutamate receptor ionotropic, NMDA; GSK-3β—Glycogen synthase kinase-3 beta; IP3R1— Inositol 1,4,5-trisphosphate receptor type 1; IP3R3—Inositol 1,4,5-trisphosphate receptor type 3; IRE1—Serine/threonine-protein kinase/endoribonuclease IRE1; miRNA—Small non-coding microRNA; NGF—Beta-nerve growth factor; PI3K—Phosphatidylinositol 3-kinase; PLCγ—1-phosphatidylinositol 4,5-bisphosphate phosphodiesterase gamma; trkA—High affinity nerve growth factor receptor; trkB—BDNF/NT-3 growth factors receptor; neurotrophic receptor tyrosine kinase 2; UPR—Unfolded protein response; XBP1—X-box-binding protein 1.

Thus, in experimental in vitro and in vivo models using Sigma1R ligands, the role of Sigma1R chaperone activation in BDNF-dependent mechanisms of antidepressant action has been demonstrated. The agonistic effect on Sigma1R can cause an increase in *Bdnf* gene expression, post-translational processing, and BDNF secretion. Sigma1R activation is involved in the regulation of trkB and associated signaling cascades, as well as the BDNF-dependent transcription factor CREB. The contribution of Sigma1R to increased phosphorylation of trkB and PLCγ might be related to the chaperone activity of Sigma1R towards trkB. Notably, the ability of Sigma1R agonists with antidepressant activity, along with normalization of the BDNF level, to restore the Sigma1R level in the brain of animals with comorbid depression has been revealed. It seems promising to study the contribution of the interaction of Sigma1R and GSK-3β to BDNF-dependent mechanisms of antidepressant action (Figure 1). The most significant cellular effects of Sigma1R agonists associated with antidepressant-like activity are shown in Table 2.

#### 5.1.2. NGF

In addition to BDNF, the neurotrophin NGF may also be involved in the Sigma1R-dependent regulation of neuroplasticity providing antidepressant action. Using PC12 cells, a concentration-dependent stimulating effect of the selective agonists of Sigma1R (+)-pentazocine (30–300 nM), imipramine (100–1000 nM), and fluvoxamine (30–3000 nM) on neurite outgrowth upon incubation in the presence of NGF (1.0 ng/mL) lasting for 48 h was demonstrated [95]. Neurosteroid DHEA (10^−11^–10^−8^ M), but not DHEAS (10^−13^–10^−4^ M), had a similar effect [103]. In the case of incubation within 5 days, DHEAS enhanced NGF-induced neurite outgrowth in PC12 cells at concentrations of 1.0 and 10 μM [93]. Under the same conditions, Sigma1R agonists 4-PPBP (0.1–10 μM), SA4503 (0.1–10 μM), and antidepressants fluvoxamine (1.0 and 10 μM), fluoxetine (1.0 and 10 μM), and escitalopram (1.0 μM) also increased the length of neurites [23,93]. The selective agonist PRE-084 (1.0 μM) increased the length of neurites upon co-incubation of PC12 cells with NGF (50 ng/mL) for 24 h [94]. However, paroxetine and sertraline had no stimulatory effect, and at a concentration of 10 μM, they showed cytotoxicity, which was more pronounced for sertraline [23,93,94]. The stimulating effect of Sigma1R ligands on neurite outgrowth at the most effective concentrations was eliminated or mitigated significantly by the Sigma1R antagonist NE–100 (1.0 μM) [23,93,94,95,103]. It is interesting that the addition of selective Sigma1R agonist PRE-084 (0.3 μM) or antagonist NE-100 (1.0 μM) to the incubation medium mitigated the cytotoxic effect of sertraline (1.0 μM). Thus, sertraline may act as inverse agonist or antagonist of Sigma1R [91,93,94]. This assumption is consistent with the clinical data [98].

Importantly, an increase of Sigma1R protein level has been established, when PC12 cells were incubated with NGF (1.0 ng/mL) in the presence of (+)–pentazocine (100 nM) or imipramine (1.0 μM). On the other hand, overexpression of the *Sigmar1* gene enhances the stimulating effect of NGF on neurite outgrowth [95]. Increased synthesis of the Sigma1R protein induces increased neuritogenesis when PC12 cells are stimulated with epidermal growth factor (EGF). Neurite outgrowth was attenuated by the Sigma1R antagonist NE-100 (1.0 μM) [197]. The results obtained suggest a positive feedback of activation of Sigma1R and neurotrophins.

Using specific inhibitors, the contribution of IP_3_R1- and NGF-associated signaling pathway proteins to the regulation of neuritogenesis of PC12 cells induced by the selective Sigma1R agonist SA4503 (1.0 μM) was established. Inhibition of IP_3_R1 by xestospongin C completely eliminated the effect of SA4503 on neurite outgrowth. Inhibitors of PLCγ, PI3K, p38MAPK, JNK, and MAPK pathways acted similarly [93]. These results are in agreement with the data on negative effect of inhibition of IP_3_R1 and PLCγ on the activity of antidepressants in vivo and in vitro [100,166,198].

With the use of Sigma1R antagonists, no contribution of the chaperone to the rapid antidepressant-like action of ketamine was demonstrated. However, NE-100 disrupted the NGF-stimulated neurite outgrowth induced by both ketamine and imipramine [101]. It is possible that the effect of ketamine on Sigma1R does not mediate a rapid antidepressant effect but contributes to the duration of the drug’s effect through the induction of neuroplasticity processes. For example, the role of IP_3_R3 in NGF-stimulated neuronal growth was revealed [199]. Sigma1R might be involved in this process by interacting with IP_3_R3 in the MAM region and stabilizing its conformation [59].

In vitro experiments demonstrated a positive effect of Sigma1R ligands with antidepressant activity on neuritogenesis in the presence of NGF, which indicates the role of the chaperone in NGF-dependent mechanisms of neuroplasticity (Table 2). The relationship between the activity of the Sigma1R chaperone and the NGF signaling pathways could contribute to the sustained antidepressant effect (Figure 1).

### 5.2. Glutamatergic Neurotransmission

A large array of data indicates an important role of the glutamatergic system in the pathogenesis of depressive disorders [15,200]. An increase in glutamate level [201], impaired receptor activity, and expression of ionotropic glutamate receptors (GluNs/NMDAr, GluAs/AMPAr) [202] have been registered in the brain structures of patients with depressive disorders. The antidepressant effect is known to be accompanied by a change in the functional activity of GluNs and GluAs [202]. The noncompetitive GluNs antagonist ketamine is of great interest in connection with the development of a rapid and sustained antidepressant effect [17].

*Sigmar1*-knockout mice are characterized by a decrease in the survival rate of newly formed neurons, inhibition of neurite outgrowth, and impaired GluN2B function in the hippocampal dentate gyrus [203], as well as a decrease in GluN2B phosphorylation in the basolateral amygdala [135]. GluNs dysfunction was accompanied by a reduction in nNOS activity and NO production. In addition, the induction of NMDAr-dependent LTP and NMDAr-independent long-term depression (LTD) is impaired in *Sigmar1*^−/−^ mice [135].

The regulation of GluNs can be explained by the protein–protein interaction of Sigma1R with the GluN1 subunit of the receptor [204] and an increase in its phosphorylation by protein kinases A and C upon ligand activation of the chaperone [205,206]. In experiments on ICR mice, the selective agonist of Sigma1R PRE-084 (3.0 nmol i.t.) and the neurosteroid DHEAS (0.3 nmol i.t.), which exhibit antidepressant-like activity, increased the level of GluN1 phosphorylation at Ser896 and Ser897 30 min after administration. Phosphorylation at these positions is known to affect the clustering of GluN1 and stimulate the transport of receptors from the ER to the cytoplasmic membrane [207]. The effect of PRE-084 and DHEAS was blocked by preliminary administration of the Sigma1R antagonist BD-1047 (100 nmol i.t.) [205,206].

Olfactory bulbectomized rats are characterized by reduced GluN1 protein level in the prefrontal cortex, hippocampus, and amygdala. Two-week administration of the selective Sigma1R agonist SA4503 (0.3 mg/kg, s.c.) caused an antidepressant-like effect, which was accompanied by restoration of the GluN1 level. The antidepressant desipramine increased GluN1 at a much higher dose (10 mg/kg, s.c.). The effect of SA4503 on the behavior of experimental animals and the level of GluN1 was eliminated by the Sigma1R antagonist NE-100 (5.0 mg/kg, s.c.) [110]. In the same experimental model of depression in mice, the phosphorylation of GluN1 and GluA1 receptors (p-GluN1 (Ser896), p-GluA1 (Ser831)) was decreased in the hippocampal dentate gyrus and CA1 region, whereas the endogenous Sigma1R agonist DHEA (30 and 60 mg/kg, p.o.) restored the phosphorylation. It has been shown that the antagonist NE-100 (1.0 mg/kg, p.o.) interfered with the action of DHEA [104,105]. The depression-like behavior of *Camk4*^−/−^ mice was also accompanied by a decrease in the level of GluA1 phosphorylation at Ser831. Preliminary administration of NE-100 (1.0 mg/kg, i.p.) eliminated the normalizing effect of SA4503 (0.3 mg/kg, p.o.) and fluvoxamine (2.5 mg/kg, p.o) on the protein phosphorylation [96].

GluNs in combination with nNOS and scaffolding protein PSD-95 form a protein complex [15]. Sigma1R regulates the interaction of the complex components, normalizes the Ca^2+^ flow through the GluN channel, and prevents excitotoxic neuronal injury by affecting nNOS [84,208]. NO plays an important role in the mechanisms of neuroplasticity and the neurobiology of depression. Therefore, maintaining NO signaling, counteraction of NO overproduction, and oxidative damage may be essential factors in antidepressant therapy [209]. In a model of depression induced by estrogen withdrawal (E2) under hormone-stimulated pregnancy, an increase in the immobilization time in the forced swim test and tail suspension test of ICR mice was combined with a decrease in the levels of nNOS, NO, and p-CREB in the hippocampus. A similar relationship was found in *Sigmar1*^−/−^ mice [125]. The antidepressant-like effects of DHEA (20 mg/kg, i.p.) and PRE-084 (1.0 mg/kg, i.p.) were concomitant with the restoration of nNOS, NO, and p-CREB levels. NMDA, a prototypical agonist of glutamate receptors, had a similar effect. The effect of DHEA on biochemical parameters was attenuated by NE-100 (1.0 mg/kg, i.p.). Experimental animals were also characterized by a decrease in the BDNF protein level and phosphorylation of GluN2B (p-NR2B). The dynamics of these changes turned out to be sensitive to the effect of NE-100 [125]. The relationship between BDNF and glutamatergic processes in the mechanisms of action of antidepressants with Sigma1R ligand properties has also been demonstrated in in vitro experiments. As was already mentioned, imipramine and fluvoxamine stimulated BDNF-dependent glutamate release in culture of cortical neurons. The action of imipramine was blocked by the Sigma1R antagonist BD-1047 [100].

Skuza and Rogoz have demonstrated a synergistic effect of GluN antagonists and Sigma1R agonists on Wistar rats in the forced swim test [128]. Antagonists of the GluNs memantine and amantadine [210] decreased the immobilization time upon combined administration with the Sigma1R agonist SA4503 at subeffective doses [128]. It must be noted that memantine and amantadine themselves, as well as the noncompetitive GluNs antagonist ketamine, have affinity for Sigma1R in the micromolar concentration range [101,211]. A recent study revealed that the rapid antidepressant-like action of ketamine and memantine (10 mg/kg, i.p.) in the original rat model of depression is accompanied by a decrease in the level of intracellular Ca^2+^ in CA1 hippocampal neurons. However, after 24 h, only ketamine caused an antidepressant-like effect, which was combined with an increase in BDNF level in the hippocampus and was eliminated by the trkB antagonist [212].

These data indicate the contribution of Sigma1R to the mechanisms of antidepressant activity through the restoration of expression and increased phosphorylation of glutamate receptors (GluNs, GluA1), as well as regulation of nNOS activity in neurons (Table 2). These processes may possibly be dependent on the interaction of Sigma1R with the GluN1 subunit (Figure 1).

### 5.3. Calcium Signaling

Calcium ions are essential in cell biology, regulating membrane excitability and acting as secondary messengers and regulators of protein activity and gene expression [213]. Calcium signaling is involved in neuroplasticity induced by BDNF and glutamatergic mechanisms [214,215,216]. The relationship between changes in the concentration of mitochondrial or intracellular calcium and the development of depressive disorders has been demonstrated [19,217,218].

Sigma1R activation induces a redistribution of intracellular calcium [40,59], which may contribute to the mechanisms of antidepressant action. The Ca^2+^ chelator EGTA (13 and 40 nmol/mouse, i.c.v.), administered 10 min before the forced swim test, had no effect on the immobilization time of the Swiss mice, but prevented dose-dependently the antidepressant-like action of the selective Sigma1R agonist igmesine, which developed 30 min after compound i.p. administration at a dose of 60 mg/kg. A similar effect was exhibited by verapamil (5 and 10 nmol, i.c.v.), a Ca_v_1 channel blocker, and a ω-conotoxin GVIA (0.1 and 0.3 nmol, i.c.v.), a blocker of Ca_v_2 channels of nerve terminals and dendrites [122]. Intracellular Ca^2+^ modulators are also able to affect the action of igmesine. Thus, the effects of igmesine were inhibited by the intracellular Ca^2+^ chelator EGTA/AM (75 and 225 nmol, i.c.v.), the IP_3_R1 antagonist xestospongin C (0.9–13.4 nmol, i.c.v.), and ryanodine at concentration activating the Ca^2+^ flow (10 nmol i.c.v.) [198]. An inductor of Ca^2+^ release from ER bradykinin (4.5 and 13.4 nmol, i.c.v.) reduced the immobilization time in mice when igmesine was administered at a subeffective dose of 30 mg/kg, i.p. The Ca_v_1 channel agonist (–)-Bay K8644 and caffeine did not alter the effect of the drug. It should be noted that chelation of Ca^2+^ by EGTA abolishes the effects of both igmesine and desipramine, which has a lower affinity for Sigma1R [109,219]. The use of EGTA/AM only neutralizes the effect of igmesine, which indicates a special role of intracellular Ca^2+^ in the antidepressant effect of Sigma1R ligand [122]. These data are consistent with the ability of EGTA (10 mM) to prevent the stimulative effect of PRE-084 on complex I (NADH-ubiquinone oxidoreductase) activity in mouse forebrain mitochondria [220].

Ca^2+^-dependent proteins play a significant role in the pathogenesis of depression. CaMKIV, being localized predominantly in the nucleus, is activated by high concentrations of Ca^2+^ and catalyzes the phosphorylation of the transcription factor CREB, enhancing the expression of BDNF and other proteins involved in neuroplasticity [25,221]. According to Moriguchi et al., knockout *Camk4* mice can be used as a depression model resistant to SSRI therapy [25,96]. Chronic administration of fluoxetine (18 mg/kg, p.o.) and paroxetine (1.0 mg/kg, p.o.) to Camk4^−/−^ mice did not cause a pronounced antidepressant-like effect and induction of neurogenesis in the hippocampal dentate gyrus [96,222]. In contrast, a two-week course of a selective agonists SA4503 (0.3 mg/kg, p.o.) or fluvoxamine (2.5 mg/kg, p.o.), which have higher affinity for Sigma1R, caused a decrease in the immobilization time of *Camk4*^−/−^ mice in the forced swim test and the tail suspension test. These effects were eliminated by preliminary administration of NE-100 at a dose of 1.0 mg/kg, i.p. The reference drug paroxetine (1.0 mg/kg, p.o.) did not affect the duration of immobilization in both tests [96].

According to Moriguchi et al., the contribution of Sigma1R to the mechanisms of antidepressant action in this experimental model may be due to the regulation of the level of intracellular Ca^2+^ and activation of an alternative CaMKIIα-dependent mechanism for controlling the expression of the *Bdnf* gene [96]. Depression-like behavior of Camk4^−/−^ mice in comparison with wild-type animals is accompanied by a combination of disturbances in the hippocampal dentate gyrus, including inhibition of neurogenesis and a decrease in the activation of BDNF signaling pathway [25,96]. A decrease in the content of phosphorylated Akt (Ser473), CaMKIIα (Thr286), and CREB (Ser133) was revealed. Moreover, a decline in the level of the Sigma1R protein, mRNAs of exons 1 and 4 of the *Bdnf* gene, and the production of ATP by mitochondria has also been demonstrated [96]. It is an interesting fact that chronic administration of SA4503 (0.3 mg/kg, p.o.) or fluvoxamine (2.5 mg/kg, p.o) augmented the values of all parameters, which, with the exception of p-CREB, reached the level of wild-type animals. The increased phosphorylation of ERK kinase at Thr202/Tyr204 was a distinctive characteristic of the action of the selective Sigma1R agonist SA4503. The key role of the Sigma1R chaperone in the mechanisms of antidepressant-like action in this experimental model was demonstrated using the Sigma1R antagonist NE-100 (1.0 mg/kg, i.p.), since preliminary administration of the drug eliminated the action of SA4503 and fluvoxamine [96]. These data are consistent with the results of experiments in olfactory bulbectomized mice, in which a decreased level of p-CaMKIIα (Thr286), but not p-CaMKIV (Thr196), was recorded in the hippocampal dentate gyrus and CA1 region. Chronic administration of DHEA increased CaMKIIα phosphorylation, while NE-100 blocked the neurosteroid action [104,105].

The chaperone activity of Sigma1R towards plasma membrane proteins can also mediate the antidepressant effect. Direct interaction between Sigma1Rs and acid-sensing ion channels 1a (ASIC1a) was demonstrated [223,224]. Ligand activation of Sigma1R promotes inhibition of ASICs and decrease in the Ca^2+^ influx into rat cortical neurons [225,226]. An antidepressant-like effect of ASIC inhibitors has been demonstrated in various experimental models [76,227]. Inhibition of ASICs upon activation of Sigma1R can possibly cause similar effects.

The above evidence indicates a significant contribution of Sigma1R to the regulation of Ca^2+^-dependent mechanisms of antidepressant action (Table 2). It is possible that activation of Sigma1R chaperone can mitigate disturbances of Ca^2+^ homeostasis, accompanying depressive disorders, by regulation of Ca^2+^ sensor proteins, Ca^2+^ permeable channels in the ER and plasma membrane, and calcium/calmodulin-dependent protein kinases (Figure 1). For more detailed information about calcium signaling pathways, see [228].

### 5.4. Unfolded Protein Response

Recent publications have discussed the role of impaired cellular proteostasis in the pathogenesis of depressive disorders [229,230,231]. The importance of chronic stress for the etiology and pathogenesis of depression is generally known [136,137]. T. Hayashi put forward a hypothesis about the transition of emotional stress into cellular stress [232]. Aversive effects on the cell trigger the ER stress, which is a set of disturbances in the maturation and packing of proteins in the ER [229]. The universal cellular response to ER stress is the activation of adaptation processes (UPR) aimed at maintaining proteostasis by reduction of protein synthesis, activation of the expression of chaperone genes, and an increase in degradation of misfolded proteins. If these adaptation mechanisms fail, the cell undergoes apoptosis [231,233]. UPR is triggered by Ca^2+^-sensitive ER stress sensors IRE1, PERK, and ATF6 upon dissociation of the BiP chaperone from them and the formation of a complex with unfolded proteins [67]. A number of proteins included in the UPR are considered promising targets for pharmacological regulation in various deleterious conditions [234].

To study the contribution of UPR signaling pathways to the pathogenesis of depression, Timberlake et al. developed an experimental model based on intrahippocampal administration of tunicamycin, which activates UPR by blocking the *N*-terminal glycosylation of newly formed proteins [235,236]. Experimental rats demonstrated pronounced depression-like behavior in a battery of tests, which was accompanied by an increase in the expression of genes associated with UPR and inflammation [235]. 

In the hippocampus of learned helpless rats, an increase of mRNAs for chaperone genes *Hspa5* (*Grp78*, *Bip*) and *Hsp90b1* (*Grp94*), the transcription activation factor *Atf6*, IRE1-dependent transcription factor *Xbp1*, PERK-dependent transcription activation factor *Atf4*, and the proapoptotic factor *Ddit* (*Chop*) was revealed [185,237]. With the exception of the *Atf4* mRNA level, similar changes were recorded in the hippocampus of rats after chronic restraint stress. It is important to emphasize the increase in both the general level of ER stress sensors IRE1 and PERK, and their phosphorylation, which indicates the activation of UPR processes [238].

Modeling of PTSD in animals (restraint stress, forced swimming, and ether exposures) provoked the UPR activation in neurons of rat’s medial prefrontal cortex. An increase in intracellular Ca^2+^ has been revealed, as well as the level of ER stress sensors and proteins of signaling cascades UPR (p-PERK/p-eIF-2a, ATF6a/XBP1, IRE1), mRNA of the *Hspa5* gene (*Grp78*, *Bip*). Increased expression of apoptosis proteins (caspase 3, 7, 12; apoptosis regulator Bax) may suggest a depletion of the UPR compensatory mechanisms [239]. In the model of depression based on chronic unpredictable mild stress in Sprague‒Dawley rats, the level of BiP and CHOP proteins increased in the hippocampus after four weeks of the experiment [240]. Similar changes were recorded after four weeks of chronic restraint stress in Swiss mice. In addition, the rise of BiP protein was found in the prefrontal cortex. The experimental animals were characterized by a lower level of BDNF in the blood serum compared to the control group [241]. Lipopolysaccharide (LPS) administration is one of the common models of depression [242]. LPS causes an increase in the *Hspa5* (*Grp78*, *Bip*) mRNA content, along with decrease in the BDNF protein in the hippocampus of Swiss albino mice [153].

The results of animal experiments are consistent with the clinical data. Patients who committed suicide, in comparison with those who died of other reasons, had increased levels of proteins BiP, GRP94, and calreticulin in the temporal cortex. The authors of the study discuss the results in terms of the relationship between ER stress and the severity of the disease and/or the effectiveness of treatment [243]. In the prefrontal cortex of MDD patients, increased levels of mRNAs of the genes *HSPA5* (*GRP78*, *BIP*), *HSP90B1* (*GRP94*), and *ATF4P3* (*ATF4*) have been detected. It is an interesting fact that differences in gene expression were found in the patients who committed suicide [244]. In leukocytes of MDD patients, increased levels of mRNAs of the genes *HSPA5* (*GRP78*, *BIP), DDIT3 (CHOP)*, and *XBP1* and the regulator of endoplasmic-reticulum-associated protein degradation (ERAD) *EDEM1* have been discovered [245].

These results provide significant evidence for the role of the UPR system proteins in the pathogenesis of depression. However, the data are discussed separately from studies showing the importance of the Sigma1R chaperone for the regulation of UPR processes. In our opinion, the contribution of the Sigma1R chaperone to the pathogenesis of depression and the pharmacodynamics of antidepressants could be related to the control of UPR signaling cascades and the maintenance of proteostasis. This assumption is consistent with the current data [40,59,63,185,186,246,247]. Experimental models have shown the relationship of the synthesis and/or change in the functional activity of the BiP chaperone (GRP78), ER stress sensor proteins (IRE1, PERK, ATF6), and transcription factors (CHOP, ATF4, XBP1) with the expression of the *Sigmar1* gene [59,63,248]. In the MAM region, Sigma1R stabilizes IRE1 and regulates its phosphorylation upon ER stress. *Sigmar1* knockdown in CHO cells decreases IRE1 phosphorylation and causes a significant increase of proapoptotic cells, which is associated with impaired IRE1-XBP1 signaling [59,63]. Overexpression of *Sigmar1* is accompanied by a decrease in stress-induced activation of PERK and ATF6 [59]. The content of mRNAs of *Hspa5* (*Grp78*, *Bip*), *Eif2ak3 (Perk)*, *Atf6*, and *Ern1 (Ire1α)* in the retina and brain of *Sigmar1*^−/−^ mice did not differ from wild-type animals [248]. However, Müller glial cells isolated from the retinas of *Sigmar1*^−/−^ mice were characterized by increased expression of the *Hspa5* (*Grp78*, *Bip*) and *Ddit* (*Chop*) genes, but decreased levels of mRNAs of *Eif2ak3 (Perk)*, *Atf6*, and *Ern1 (Ire1α)* [248].

In light of the contribution of Sigma1R to the regulation of UPR, the possibility of enhancing BDNF synthesis due to chaperone interaction with the ER stress sensor IRE1 is necessary to emphasize. In primary mouse hippocampal neurons, glutamate-dependent induction of the IRE1‒XBP1 signaling pathway in distal dendrites promoted the expression of the *Bdnf* in the body of neurons. It has also been revealed that the *Bdnf* gene expression can be enhanced upon activation of the IRE1‒XBP1 pathway by neurotrophin itself [64,249]. The ability of the Sigma1R chaperone to stabilize IRE1 in the MAM region and activate the XBP1 transcription factor [63] may contribute to the enhancement of BDNF expression and mediate the antidepressant effect of Sigma1R agonists.

Recently, a crucial role of Sigma1R for IRE1-dependent production of proinflammatory cytokines has been established [250]. It was found that ER stress–induced IRE1 activation can mediate the cross-talk of GSK-3β and XBP-1 to differentially regulate proinflammatory cytokine production. GSK-3β activation inhibited XBP-1 splicing, resulting in the downregulation of TNF-α production [251]. These data align with the above-mentioned decrease of GSK-3β inhibitory phosphorylation at Sigma1R activation [105]. It was demonstrated that upon ER stress, an increase of unbounded BiP content in ER lumen also promotes GSK-3β activation [252].

The ability of LPS to activate IRE1 endonuclease activity, mRNA splicing of the XBP1 transcription factor, and its expression was demonstrated. *Sigmar1*^−/−^ mouse bone marrow macrophages exhibited increased inducible, but not basal, endonuclease activity of IRE1 and produced an elevated level of the IL6 and pro-IL1β transcripts. On the other hand, overexpression of the chaperone gene causes an inverse effect on the production of proinflammatory cytokines. It should be emphasized that the Sigma1R ligand antidepressant fluvoxamine (20 mg/kg, i.p.) has a protective effect in animal models of inflammation [250]. Fluvoxamine (10 μg/mL) increased the level of mRNA and Sigma1R protein in Neuro2a cells after 12 and 6 h of incubation, respectively [189]. Elevated expression of the *Sigmar1* gene by fluvoxamine was combined with activation of the *Atf4* gene, which was blocked by the selective Sigma1R antagonist NE-100 (1.0 μg/mL) or did not develop in the culture of *Sigmar1*^−/−^ fibroblasts. Fluvoxamine, but not paroxetine, inhibited apoptosis of Neuro2a cells under tunicamycin-emulated ER stress. This cytoprotective effect was also abolished by NE-100 or mitigated significantly in a culture of *Sigmar1*^−/−^ fibroblasts. At the same time, fluvoxamine did not affect the ATF6α processing, Xbp1 splicing, or BiP protein level; however, it reduced the level of the proapoptotic transcription factor CHOP. The effect of the drug did not depend on PERK. This study indicates that the cytoprotective effect of fluvoxamine potentially depends on an increase of ATF4 translation and that enhancement of *Sigmar1* expression, however, does not affect the activity of ER stress sensors [189]. 

It is reasonable to compare the effect of fluvoxamine with the effect of the prototype agonist Sigma1R (+)-pentazocine upon cellular stress. RGC-5 mouse neuronal precursor cells responded to oxidative stress under 24 h incubation in the xanthine–xanthine oxidase system by predominantly increasing the synthesis of mRNAs of *Hspa5* (*Grp78*, *Bip*), *Eif2ak3 (Perk)*, *Atf6*, *Ern1 (Ire1α)*, and *Ddit* (*Chop*), but with a decrease in the *Sigmar1* mRNA level. After 6 h of incubation in the culture, the level of the Sigma1R‒BiP complex rose, and later, after 18 h, the elevation of Sigma1R phosphorylation was detected [192]. Treatment with (+)-pentazocine (3.0 μM) caused a decline in *Hspa5* (*Grp78*, *Bip*), *Eif2ak3 (Perk)*, *Atf6*, *Ern1 (Ire1α)*, and *Ddit* (*Chop*) genes expression, and an increase in the *Sigmar1* mRNA level after 2 h of incubation. Selective agonist of Sigma1R (+)-pentazocine blocked the stimulatory effect of the xanthine–xanthine oxidase system on the formation of the Sigma1R‒BiP complex and phosphorylation of Sigma1R [192]. Oxidative stress, induced by H_2_O_2_ treatment of human lens cell culture FHL124, caused an increase of BiP, ATF6, and p-eIF-2α proteins [253]. Phosphorylation of the translation initiation factor eIF-2α is accompanied by its inhibition and reflects the activation of the PERK-dependent pathway of UPR [254]. After 24 h of incubation with H_2_O_2_ (30 μM), the Sigma1R protein level in FHL124 cells was elevated by 2.5 times. H_2_O_2_-treated cells exposed to (+)-pentazocine (10 μM) demonstrated the amount of BiP equal to control values (after 4 h) and significantly reduced level of p-eIF-2α [253]. Recapitulating the results of the studies, we can emphasize an increase in the expression of the main chaperone ER *Hspa5* (*Grp78*, *Bip*) and the proapoptotic transcription factor *Ddit* (*Chop*) genes in *Sigmar1*^−/−^ mice when modeling depression in vivo and upon induction of oxidative stress in vitro. Additionally, the antidepressant fluvoxamine and the prototype agonist Sigma1R (+)-pentazocine evoked the decrease of *Ddit* (*Chop*) expression and augmentation of *Sigmar1* expression but had a heterogeneous effect on the UPR signaling cascades and the BiP level in vitro.

Thus, current data demonstrate a substantial contribution of UPR processes to the pathogenesis of depression. The agonistic effect on Sigma1R ensures the regulation of ER stress sensors, activation of transcription factors, increased expression of *BDNF* gene, antioxidant defense proteins, and chaperones (Table 2). The combination of these processes probably contributes to the survival of neurons in target areas of the brain and the development of antidepressant action (Figure 1).

## 6. Sigma1R Chaperone and the Epigenetic Regulations in Antidepressant Action

A large number of proteins are involved in the epigenetic regulation of gene expression under stress-related disorders [255], and their function, in certain circumstances, depends on the activity of chaperones [256]. The interaction of chaperones with histones in the processes of assembly, functioning, and degradation of nucleosomes has been demonstrated [257]. The effect of Sigma1R on gene expression, demonstrated in a number of studies [258,259,260], suggests the implication of Sigma1R to the control of epigenetic mechanisms of antidepressant action. Cocaine has been shown to act agonistically on Sigma1R and cause chaperone translocation towards the nuclear membrane and the formation of a chromatin remodeling complex (emerin/HDAC/BAF). This protein complex binds to the *Maob* gene promoter and suppresses its expression [75]. The results obtained in vitro correspond to the neuroprotective effect of Sigma1R agonists in modeling Parkinson’s disease in vivo [261,262] and the effectiveness of MAO-B inhibitors in comorbid depressive disorders [263].

The pharmacodynamics of many antidepressants is associated with epigenetic processes by influencing histone modification, DNA methylation, and noncoding RNAs (miRNAs) [190,264,265]. In particular, the epigenetic effects of the Sigma1R SSRI ligand fluoxetine were revealed in the social defeat stress model. The drug restores histone acetylation (H3K14) in the hippocampus of mice after social defeat stress. An antidepressant-like effect in this model was evoked by intrahippocampal infusion of an HDAC inhibitor [266]. Increased binding of the transcription factor ΔFosB to the *Camk2a* promoter in the nucleus accumbens (NAc) of experimental animals was shown. Chronic administration of fluoxetine (20 mg/kg, i.p.) caused a decrease in acetylation and an increase in demethylation of H3K9 in the *Camk2a* promoter, which was accompanied by reduced binding of ΔFosB to the promoter followed by a decreased expression of the gene. Overexpression of the *Camk2a* gene in the NAc abolished the effect of fluoxetine in the social defeat stress model [267]. It should be noted that the Sigma1R agonist cocaine induces *Camk2a* in the NAc through binding of ΔFosB to the gene promoter [268]. Thus, the participation of Sigma1R in the epigenetic effects of fluoxetine is controversial, and the effect of drugs on Sigma1R requires further evaluation [91,92]. Another SSRI with affinity for Sigma1R citalopram altered DNA methylation of the promoters of genes associated with depressive disorders, including *TSPO* and *BDNF* in vitro [191]. A normalizing effect of chronic fluoxetine administration on the level of mir-451 in the hippocampus of rats subjected to stress at an early age (maternal separation) was revealed. Bioinformatic analysis indicates the contribution of miR-451 to the regulation of genes associated with the CREB transcription factor, GABAergic and cholinergic neurotransmission [190]. 

Recent studies have uncovered the importance of Sigma1R in the modulation of mir-214-3p, the level of which is increased in *Sigmar1*^−/−^ cells of the retina of rd10 mice [193] and the prefrontal cortex of mice after chronic social defeat stress [194]. Mir-214-3p reduces β-catenin expression [194], while mir-214 is involved in the regulation of peroxidation processes [195,196]. Selective agonist of Sigma1R (+)-pentazocine (0.5 mg/kg, i.p.), which has an antidepressant-like effect [30,121], decreased the level of mir-214-3p in retinal cells [193]. Interestingly, the regulatory effect of Sigma1R on mir-214-3p is consistent with the activity of endogenous agonist Sigma1R DHEA (60 mg/kg, p.o.), the course of which increased the count of β-catenin-positive neurons in the hippocampus of mice with olfactory bulbectomy. DHEA’s effect was attenuated by the Sigma1R antagonist NE-100 (1.0 mg/kg, p.o.) [105]. The contribution of the mir-214-3p in the nucleus accumbens (NAc) to both the pathogenesis of depression and the pharmacodynamics of escitalopram was revealed in chronic unpredictable mild stress model in rats [269]. Taking into account the significance of Sigma1R for the mechanisms of response to ER stress (UPR) [247] and the implication of UPR processes to the pathogenesis of depression [235], the decreased expression of mir-214 and enhanced production of the transcription factor XBP1 are of interest upon thapsigargin- or tunicamycin-elicited ER stress in tumor cell culture [270]. These changes could arise from the activation of the Sigma1R chaperone or distinctions of epigenetic regulation by miRNAs in tumor and normal cells.

Thus, the latest studies demonstrate the role of Sigma1R in epigenetic processes that promote antidepressant action by inducing the expression of BDNF, TSPO, and CaMKIIα, proteins that ensure the functioning of neurotransmitter systems (Figure 1, Table 2).

## 7. Conclusions

In summary, current data demonstrate that the Sigma1R chaperone interacts with previously identified cellular mechanisms, which are associated with the formation of a depressive phenotype. Sigma1R is also involved in the pharmacodynamic mechanisms of antidepressants with various pharmacological targets. Therefore, despite the lack of direct antidepressant action of Sigma1R ligands, pharmacological activation of this chaperone can be considered a promising strategy to improve and develop approaches for combined, adjuvant pharmacotherapy of depression.

## Figures and Tables

**Table 1:** Sigma1R ligand with antidepressant properties.

Compound	Sigma1R Activity	Sigma1R Affinity, Ki
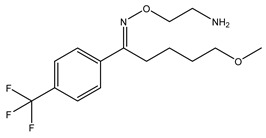 Fluvoxamine	Agonist [23,26,31,93,94,95,96]	36.0 nM [21] 17.0 nM [23]
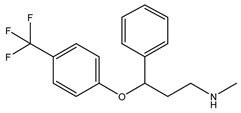 Fluoxetine	Putative agonist [23,91,92,93,97]	240.0 nM ^1^ [21] 191.2 nM [23]
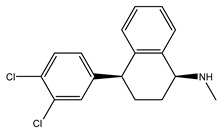 Sertraline	Putative antagonist Putative inverse agonist [91,92,93,94,98]	57.0 nM [21] 31.6 nM [23]
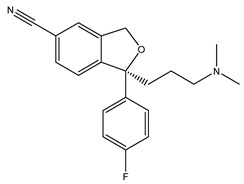 Escitalopram	Agonist [23,93]	288.3 nM [23]
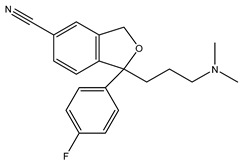 Citalopram	Agonist [99]	292.0 nM [21] 403.8 nM [23]
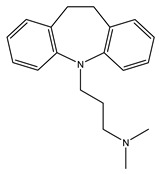 Imipramine	Agonist [95,100,101,102]	343.0 nM [21] 332.1 nM [101]
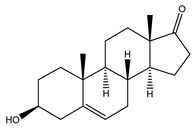 DHEA	Agonist [103,104,105]	2.96 μM [106] 706.0 nM [107]
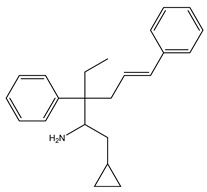 Igmesine (JO-1784)	Agonist [92,108]	75.0 nM [109]
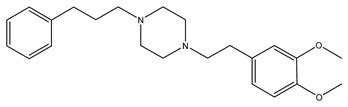 Cutamesine (SA4503)	Agonist [93,110]	17.4 nM ^2^ [111]
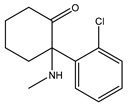 Ketamine	Putative agonist [101,112]	139.6 μM ^3^ [101]

1 Ki for (±)-fluoxetine; 2 IC_50_ 3 Ki for (±)-ketamine.

**Table 2:** The effects of Sigma1R agonists associated with antidepressant-like activity.

Sigma1R Agonists	Effects	
Fluvoxamine	BDNF	Upregulation of Akt1 phosphorylation (Ser473) in vitro [177]. Increases proBDNF and mBDNF levels in the hippocampus and prefrontal cortex of rats with comorbid depression-like disorder. The effects were blocked by Sigma1R antagonist NE-100 [31].
	NGF	Stimulates neurite outgrowth upon incubation in the presence of NGF in vitro [23,93,94,95]. The effect was blocked by Sigma1R antagonist NE-100 [93,94].
	Calcium signaling	Causes a decrease in the immobilization time of *Camk4*^−/−^ mice in the forced swim test and the tail suspension test. The effect was blocked by Sigma1R antagonist NE-100 [96].
	ER stress and unfolded protein response	Increases expression of the *Sigmar1* and the *Atf4* genes in vitro. Inhibits apoptosis of Neuro2a cells under simulated ER stress. The effects were blocked by Sigma1R antagonist NE-100 [189].
Fluoxetine	NGF	Stimulates neurite outgrowth upon incubation in the presence of NGF in vitro [23].
	Epigenetic regulation of gene expression	Normalizes the level of mir-451 in the hippocampus of rats subjected to stress at an early age (maternal separation) [190].
Escitalopram	NGF	Stimulates neurite outgrowth upon incubation in the presence of NGF in vitro [23].
Citalopram	Epigenetic regulation of gene expression	Alters the methylation of the promoter regions of *TSPO* and *BDNF* genes in vitro [191].
Imipramine	BDNF	Enhances BDNF-induced glutamate release and intracellular Ca^2+^ increase in vitro. Stimulates of PLCγ binding to trkB and PLCγ phosphorylation in vitro. The effects were blocked by Sigma1R antagonist BD-1047 [100].
	NGF	Stimulates neurite outgrowth upon incubation in the presence of NGF in vitro [95,101]. The effect was blocked by Sigma1R antagonist NE-100 [101].
DHEA	BDNF	Increases Akt1 phosphorylation (Ser473) in vitro [177]. Reduces the level of pGSK-3β (Ser9) in the hippocampal dentate gyrus of olfactory bulbectomized mice. The effect was blocked by Sigma1R antagonist NE-100 [105].
	NGF	Stimulates neurite outgrowth upon incubation in the presence of NGF in vitro [103].
	Glutamatergic neurotransmission	Restores the phosphorylation of GluN1 (Ser896) and GluA1 (Ser831) receptors in the hippocampal dentate gyrus and CA1 region of olfactory bulbectomized rats. The effect was blocked by Sigma1R antagonist NE-100 [104,105]. Restores levels of nNOS, NO, and p-CREB in the hippocampus of ICR mice in a model of depression induced by estrogen withdrawal (E2) under hormone-stimulated pregnancy. The effect was attenuated by Sigma1R antagonist NE-100. Experimental animals were characterized by a decrease in the BDNF protein level and phosphorylation of GluN2B (p-NR2B) in the hippocampus. The dynamics of these changes was sensitive to the effect of Sigma1R antagonist NE-100 [125].
	Calcium signaling	Increases CaMKIIα phosphorylation in the hippocampus of olfactory bulbectomized mice. The effect was blocked by Sigma1R antagonist NE-100 [104,105].
Igmesine (JO-1784)	Calcium signaling	The antidepressant-like effect of igmesine was prevented by Ca^2+^ channel blockers [122].
Cutamesine (SA4503)	BDNF	Increases BDNF secretion in vitro [161]. Increase in the BDNF content in the hippocampus of naïve rats [163].
	NGF	Stimulates neurite outgrowth upon incubation in the presence of NGF in vitro. The effect was blocked by Sigma1R antagonist NE-100 [93].
	Glutamatergic neurotransmission	Restoration of the GluN1 level in the prefrontal cortex, hippocampus, and amygdala of olfactory bulbectomized rats. The effect was blocked by Sigma1R antagonist NE-100 [110].
	Calcium signaling	Causes a decrease in the immobilization time of *Camk4*^−/−^ mice in the forced swim test and the tail suspension test. The effect was blocked by Sigma1R antagonist NE-100 [96].
(+)-pentazocine	NGF	Stimulates neurite outgrowth upon incubation in the presence of NGF in vitro [95].
	ER stress and unfolded protein response	Causes a decrease in the expression of the genes *Hspa5* (*Grp78*, *Bip*), *Eif2ak3 (Perk)*, *Atf6*, *Ern1 (Ire1α)*, and *Ddit* (*Chop*), and an increase in the *Sigmar1* mRNA upon cellular stress in vitro. Blocks the stimulatory effect of the xanthine–xanthine oxidase system on the formation of the Sigma1R‒BiP complex and phosphorylation of Sigma1R [192].
	Epigenetic regulation of gene expression	Decreases the level of mir-214-3p in retinal cells [193]. Mir-214-3p reduces β-catenin expression [194], while mir-214 is involved in the regulation of peroxidation processes [195,196].
(+)-SKF-10.047	BDNF	Increases BDNF secretion in vitro. The effect was blocked by Sigma1R antagonist BD-1063 [162].
PRE-084	BDNF	Increases neurite outgrowth in vitro due to the phosphorylation of BDNF trkB receptors. The effect was blocked by Sigma1R antagonist BD-1063 [165]. Increased the level of pGSK-3β (Ser9) in the hippocampus of naïve mice [127].
	NGF	Stimulates neurite outgrowth upon incubation in the presence of NGF in vitro. The effect was blocked by Sigma1R antagonist NE-100 [94].
	Glutamatergic neurotransmission	Restoration of nNOS, NO, and p-CREB levels in the hippocampus of ICR mice in a model of depression induced by estrogen withdrawal (E2) under hormone-stimulated pregnancy [125].
Ketamine	NGF	Stimulates neurite outgrowth upon incubation in the presence of NGF in vitro. The effect was blocked by Sigma1R antagonist NE-100 [101].

## Abbreviations

5-HT	5-hydroxytryptamine; serotonin
Akt	AKT serine/threonine kinase
Akt1	RAC-alpha serine/threonine-protein kinase
AMPA	2-amino-3-(5-methyl-3-oxo-2,3-dihydro-1,2-oxazol-4-yl)propanoic acid
ASIC	Acid-sensing ion channel
ATF4	Cyclic AMP-dependent transcription factor ATF-4
ATF6	Cyclic AMP-dependent transcription factor ATF-6 alpha
BDNF *BDNF*	Brain-derived neurotrophic factor Human BDNF gene
*Bdnf*	Rodent BDNF gene
BiP	Endoplasmic reticulum chaperone BiP
CaMKIIα	Calcium/calmodulin-dependent protein kinase II alpha
CaMKIV	Calcium/calmodulin-dependent protein kinase type IV
CHOP	DNA damage-inducible transcript 3 protein
CRAC/CARC	Cholesterol recognition/interaction amino acid consensus
CREB	Cyclic AMP-responsive element-binding protein
CRF	Corticoliberin; Corticotropin releasing factor
DHEA	Dehydroepiandrosterone
DHEAS	Dehydroepiandrosterone sulfate
EDEM	ER degradation-enhancing alpha-mannosidase-like protein 1
EGTA	Ethylene glycol-bis(2-aminoethylether)-N,N,N’,N’-tetraacetic acid
eIF-2a	Eukaryotic translation initiation factor 2
ER	Endoplasmic reticulum
ERK1	Mitogen-activated protein kinase 3; extracellular-signal-regulated kinase 1
ERK2	Mitogen-activated protein kinase 1; extracellular signal-regulated kinase 2
GluA	Glutamate receptor ionotropic, AMPA
GluN	Glutamate receptor ionotropic, NMDA
GRP94	Endoplasmin
GSK-3β	Glycogen synthase kinase-3 beta
HPA	Hypothalamic-pituitary-adrenal axis
IP3R1	Inositol 1,4,5-trisphosphate receptor type 1
IP3R3	Inositol 1,4,5-trisphosphate receptor type 3
IRE1	Serine/threonine-protein kinase/endoribonuclease IRE1
JNK	C-Jun *N*-terminal kinase; mitogen-activated protein kinase
LPO	Lipid peroxidation
LPS	Lipopolysaccharide
LTD	Long-term depression
LTP	Long-term potentiation
MAM	Mitochondria-associated membranes
mBDNF	Mature brain-derived neurotrophic factor
miRNA	Small non-coding microRNA
MDD	Major depressive disorder
nNOS	Nitric oxide synthase, brain
NGF	Beta-nerve growth factor
NMDA	(2R)-2-(methylamino)butanedioic acid
NO	Nitric oxide
P35	Cyclin-dependent kinase 5 activator 1
p38MAPK	p38 mitogen-activated protein kinase
PDGFRβ	Platelet-derived growth factor receptor beta
PERK	Eukaryotic translation initiation factor 2-alpha kinase 3
PI3K	Phosphatidylinositol 3-kinase
PKC	Protein kinase C
PLCγ	1-phosphatidylinositol 4,5-bisphosphate phosphodiesterase gamma
proBDNF	Precursor of brain-derived neurotrophic factor
PTSD	Post traumatic stress disorder
Rac1-GTPase	Rat sarcoma virus mitogen activated protein kinase kinase
SERT	Sodium-dependent serotonin transporter
Sigma1R	Sigma nonopioid intracellular receptor 1; chaperon Sigma1R
SIGMAR1	Human Sigma1R gene
Sigmar1	Rodent Sigma1R gene
SSRI	Selective serotonin reuptake inhibitor
trkA	High affinity nerve growth factor receptor
trkB	BDNF/NT-3 growth factors receptor; neurotrophic receptor tyrosine kinase 2
TSPO	Translocator protein
UPR	Unfolded protein response
VDAC2	Voltage-dependent anion-selective channel protein 2
XBP1	X-box-binding protein 1
